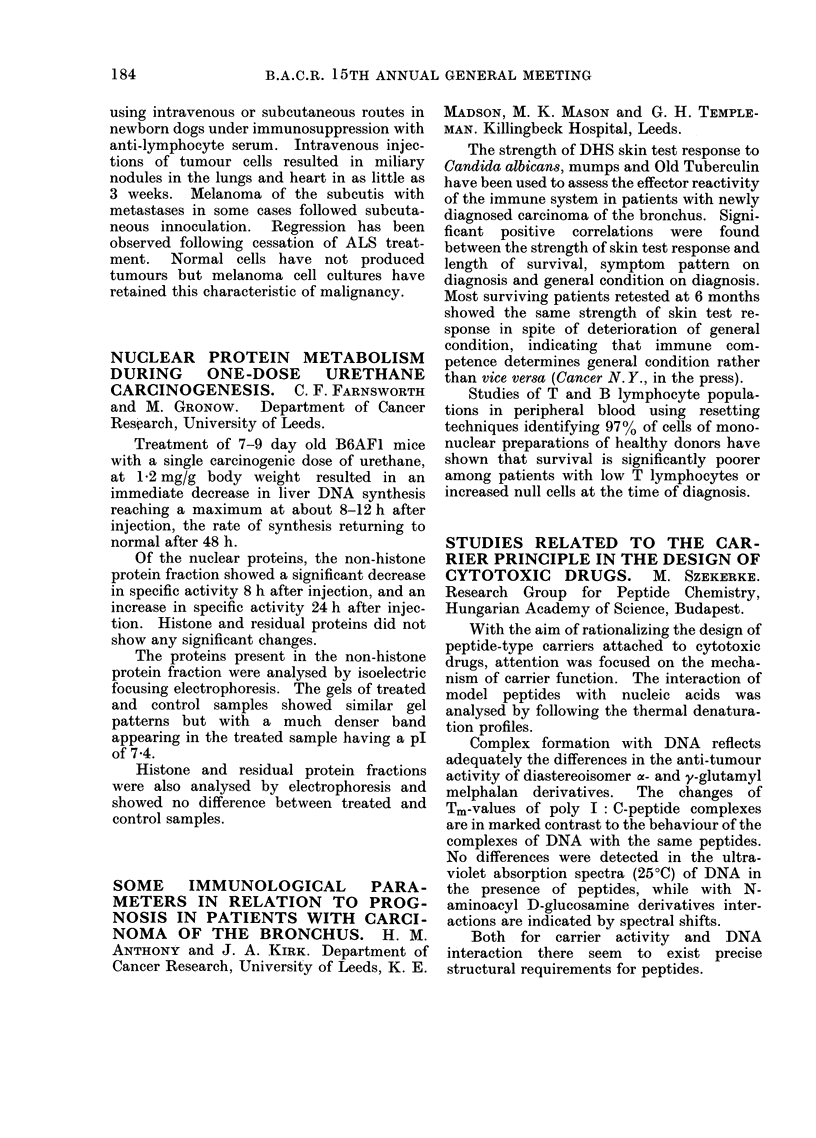# Proceedings: Some immunological parameters in relation to prognosis in patients with carcinoma of the bronchus.

**DOI:** 10.1038/bjc.1974.170

**Published:** 1974-08

**Authors:** H. M. Anthony, J. A. Kirk, K. E. Madson, M. K. Mason, G. H. Templeman


### SOME IMMUNOLOGICAL PARAMETERS IN RELATION TO PROGNOSIS IN PATIENTS WITH CARCINOMA OF THE BRONCHUS.

H. M. Anthony and J. A. Kirk. Department of Cancer Research, University of Leeds, K. E. Madson, M. K. Mason and G. H. Templeman. Killingbeck Hospital, Leeds.

The strength of DHS skin test response to *Candida albicans*, mumps and Old Tuberculin have been used to assess the effector reactivity of the immune system in patients with newly diagnosed carcinoma of the bronchus. Significant positive correlations were found between the strength of skin test response and length of survival, symptom pattern on diagnosis and general condition on diagnosis. Most surviving patients retested at 6 months showed the same strength of skin test response in spite of deterioration of general condition, indicating that immune competence determines general condition rather than *vice versa* (*Cancer N.Y.*, in the press).

Studies of T and B lymphocyte populations in peripheral blood using resetting techniques identifying 97% of cells of mononuclear preparations of healthy donors have shown that survival is significantly poorer among patients with low T lymphocytes or increased null cells at the time of diagnosis.